# Supplementary material for: Identification of novel fructo-oligosaccharide bacterial consumers by pulse metatranscriptomics in a human stool sample
Source: mSphere. 2024 Dec 19;10(1):e00668-24. doi: 10.1128/msphere.00668-24 (PMC11774028; doi:10.1128/msphere.00668-24)
Supplement: Supplemental Material — Figures S1-S3; Tables S5 and S6. [file msphere.00668-24-s0001.pdf]

Supplementary Information

Identification of novel fructo-oligosaccharide bacterial consumers by pulse metatranscriptomics in a human stool sample

Catherine Prattico[a], Emmanuel Gonzalez[b,c,d], Lharbi Dridi[e], Shiva Jazestani[e], Kristin E. Low[f], D. Wade Abbott[f], Corinne F. Maurice*[a,g], Bastien Castagner*[eg]

[a]Department of Microbiology & Immunology, Faculty of Medicine and Health Sciences, McGill University, 845 Sherbrooke St. W., Montréal, Québec, H3A 0G4 Canada.

[b]Canadian Centre for Computational Genomics, McGill Genome Centre, McGill University, 740 Dr Penfield Ave, Montréal, Québec, H3A 0G1, Canada.

[c]Department of Human Genetics, McGill University, 740 Dr Penfield Ave, Montréal, Québec, H3A 0G1, Canada.

[d]Gerald Bronfman Department of Oncology, McGill University, 5100 de Maisonneuve Blvd. West, Montréal, Québec, H4A 3T2, Canada

[e]Department of Pharmacology & Therapeutics, Faculty of Medicine and Health Sciences, McGill University, 3655 Prom. Sir-William-Osler, Montréal, Québec, H3G 1Y6, Canada

[f]Agriculture and Agri-Food Canada, Lethbridge Research and Development Centre, Lethbridge, Alberta, T1J 4B1, Canada

[g]McGill Centre for Microbiome Research, McGill University, 3775 Rue University, Montréal, Québec, H3A 2B4, Canada.

*correspondence: bastien.castagner@mcgill.ca & corinne.maurice@mcgill.ca

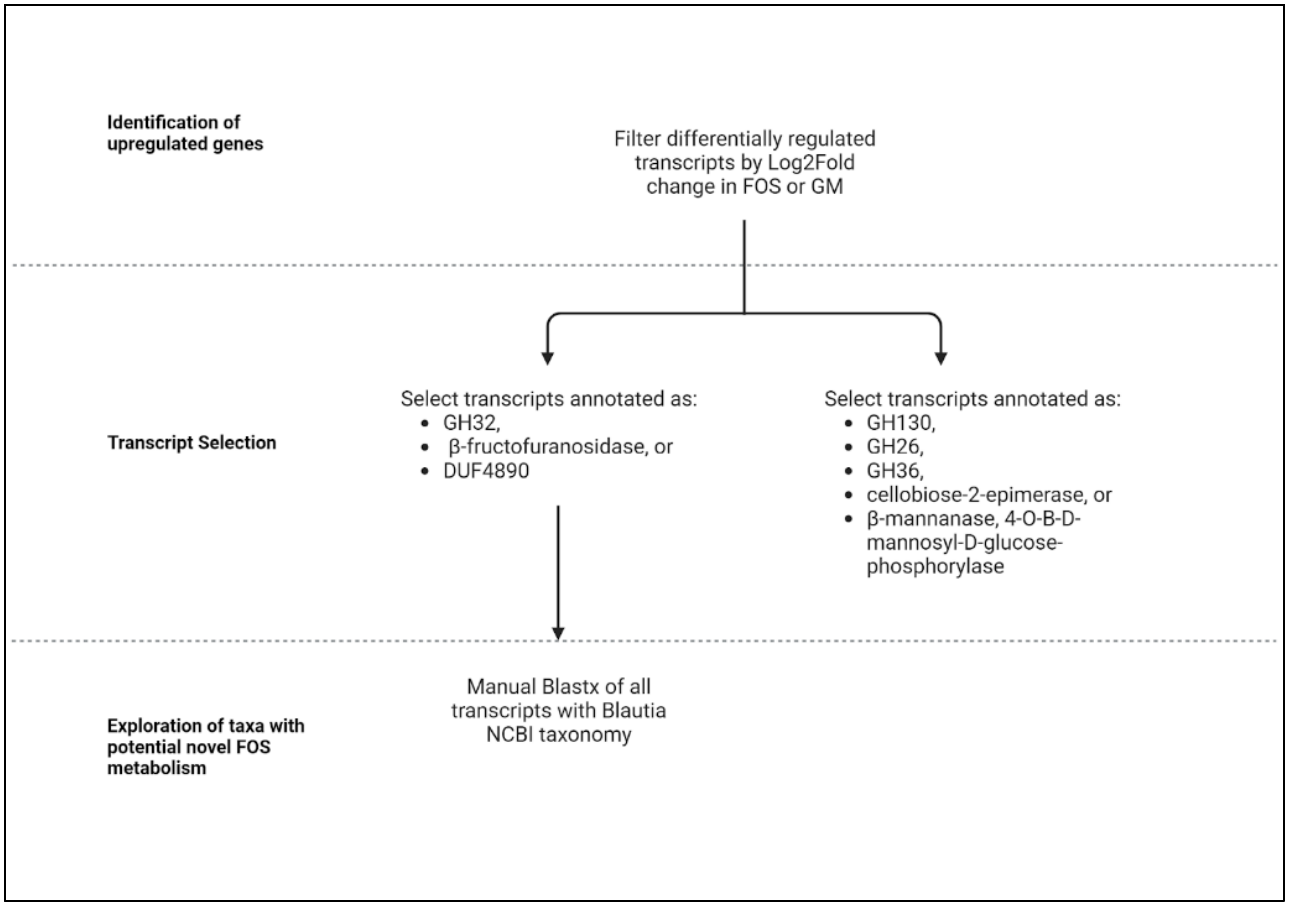

Figure S1. Workflow of RNAseq transcript selection process.

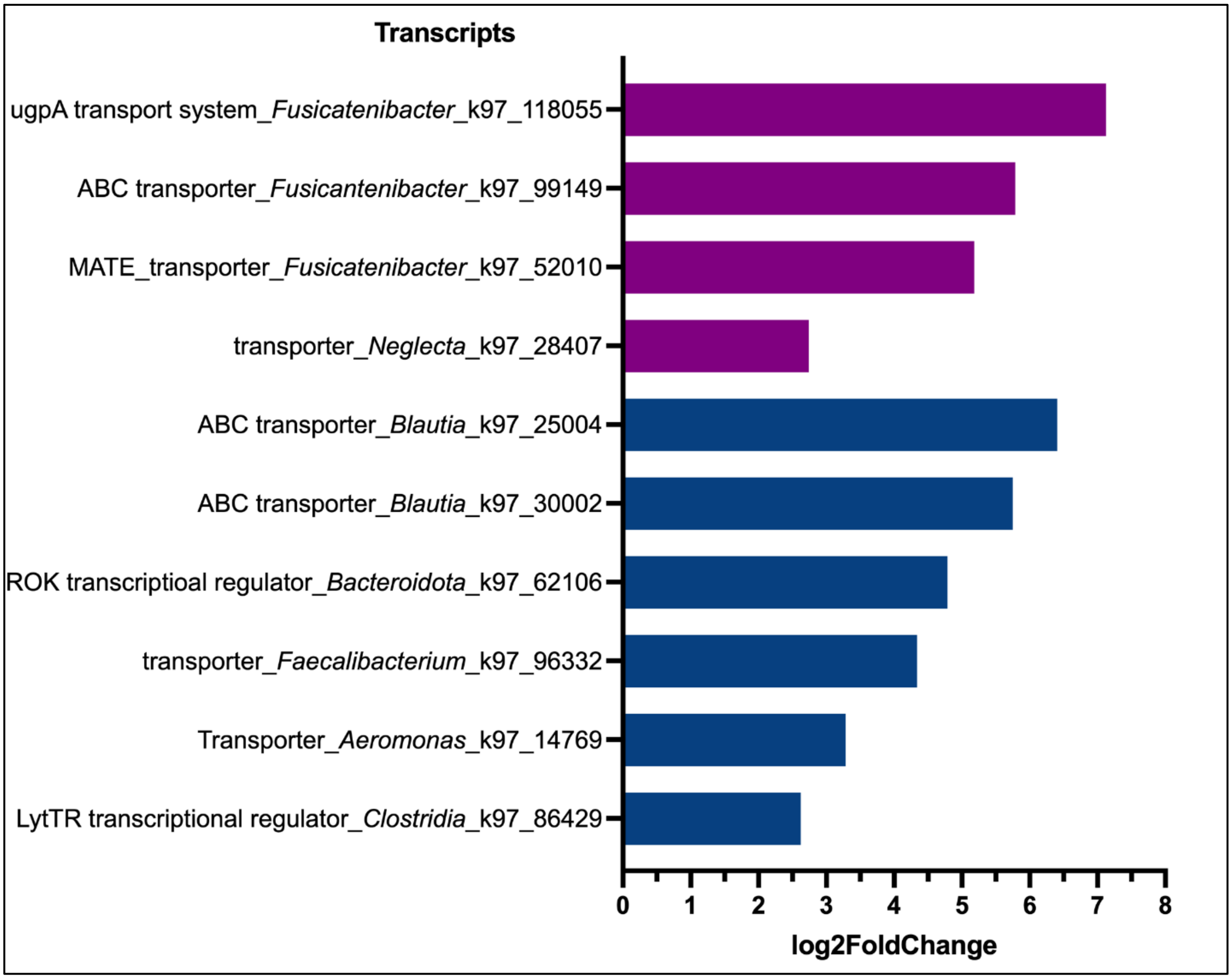

Figure S2. RNASeq differential abundance of transcripts containing transporters or transcriptional regulators related to FOS (blue) or galactomannan (purple) metabolism.

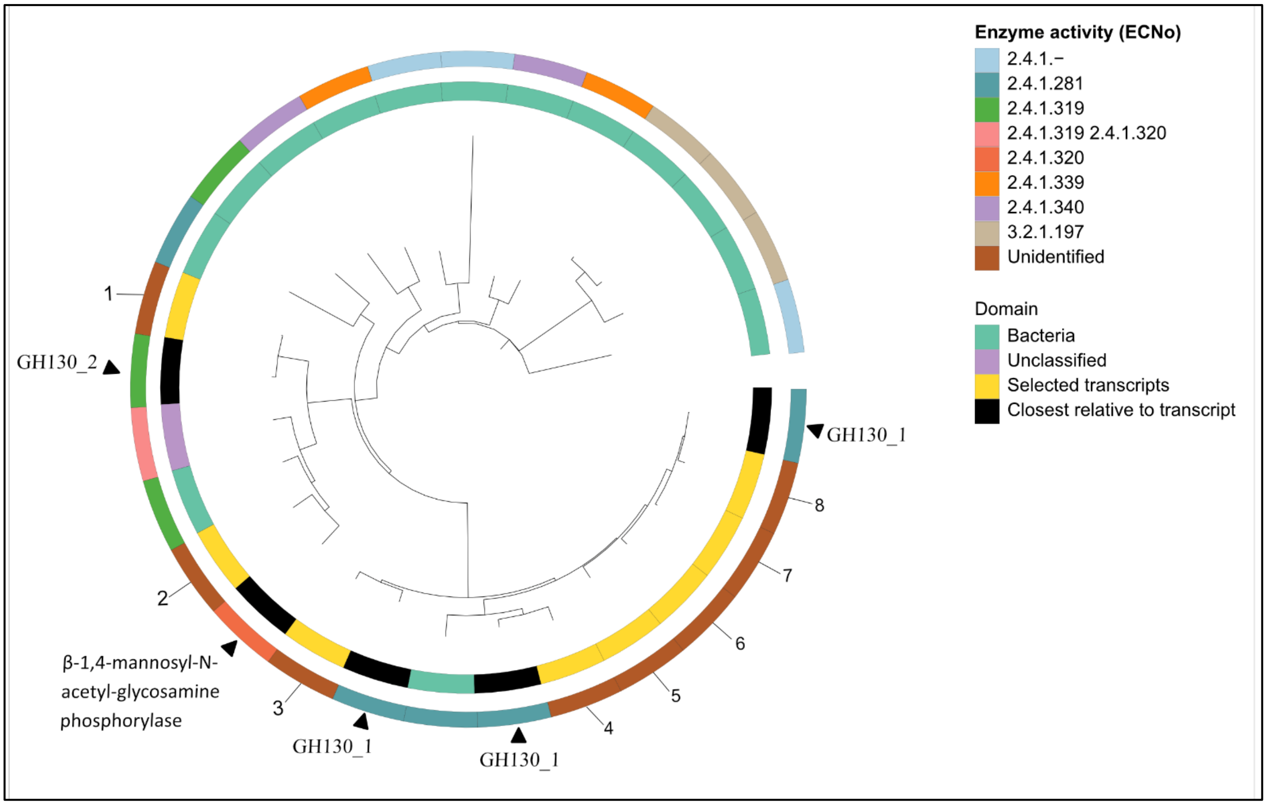

Figure S3. SACCHARIS analysis of GH130 predicted enzyme substrate affinity. The phylogenetic trees were generated using the SACCHARIS 2.0 pipeline. The enzyme origin (bacteria (turquoise), unclassified (purple), or our transcript sequences (yellow)) is depicted in the inner circle. The closest neighbouring sequences are depicted in black and are active on β-mannans ((GH130__1; β-1,4-mannosylglucose phosphorylase; 2.4.1.281), (GH130_2; β-1,4-manooligosaccharide phosphorylase; 2.4.1.319)) or on N-glycans (β-1,4-mannosyl-*N*-acetyl-glucosamine phosphorylase; 2.4.1.320). The enzyme activity (ECNo) is shown in the outer circle.

Table S5. Strains used in this study.

Strain	Source	Distributer	% Identity to ANCHOR V4V5	% Identity to ANCHOR V5V6
Blautia faecicola DSM 107827	Human faeces	DSMZ GmbH	NA	NA
Blautia faecis DSM 27629	Human faeces	DSMZ GmbH	100.0	100.0
Blautia luti DSM 14534	Human faeces	DSMZ GmbH	100.0	100.0
Blautia massiliensis DSM 101187	Human faeces	DSMZ GmbH	100.0	100.0
Blautia obeum DSM 25238	Human faeces	DSMZ GmbH	100.0	100.0
Blautia wexlerae DSM 19850	Human faeces	DSMZ GmbH	100.0	100.0

Table S6. Difference in area under the curve (ΔAUC) between MMe alone growth curve and each growth curve of MMe supplemented with 0.5% glucose, 0.5% FOS, 0.5% inulin, and 0.5% levan. Dunnet test was performed with glucose as a control to compare growth in different medium conditions. ** indicate $p<0.005$; *** indicate $p<0.0005$

Blautia species	Enriched Minimum Medium			
	+ glucose	+ FOS	+ inulin	+ levan
faecicola	+ 99.17	+14.92 (*** p < 0.0001)	+15.47 (*** p < 0.0001)	-2.09 (** p = 0.0030)
faecis	+17.20	+25.92 (p = 0.1236)	+23.95 (p = 0.2579)	-3.14 (** p < 0.0027)
luti	+34.85	+4.40 (*** p < 0.0001)	-0.44 (*** p < 0.0001)	-0.26 (*** p < 0.0001)
massiliensis	+34.72	+32.95 (p = 0.7819)	+30.93 (p = 0.1702)	-1.93 *** (p <0.0001)
